# The “sugar‐coated bullets” of cancer: Tumor‐derived exosome surface glycosylation from basic knowledge to applications

**DOI:** 10.1002/ctm2.204

**Published:** 2020-10-13

**Authors:** Shanyi Lin, Shumin Zhou, Ting Yuan

**Affiliations:** ^1^ Department of Orthopaedic Surgery Shanghai Jiao Tong University Affiliated Sixth People's Hospital Shanghai P. R. China; ^2^ Institute of Microsurgery on Extremities Shanghai Jiao Tong University Affiliated Sixth People's Hospital Shanghai P. R. China

**Keywords:** biomarkers, exosomes, glycosylation, purification, targeting therapy

## Abstract

Scientific interest in exosomes has exploded in recent decades. In 1990 only three articles were published on exosomes, while over 1,700 have already been published half‐way into 2020.^1^ While researchers have shown much interest in exosomes since being discovered in 1981, an appreciation of the potential role of glycans in exosome structure and function has emerged only recently. Glycosylation is one of the most common post‐translational modification, which functions in many physiological and pathological aspects of cellular function. Many components of exosomes are heavily glycosylated including proteins, lipids, among others. Thus, glycosylation undoubtedly has a great impact on exosome biosynthesis and function. Despite the importance of glycosylation in exosomes and the recent recognition of them as biomarkers for not only malignancies but also other system dysfunction and disease, the characterization of exosome glycans remains understudied. In this review, we discuss glycosylation patterns of exosomes derived from various tissues, their biological features, and potential for various clinical applications. We highlight state‐of‐the‐art knowledge about the fine structure of exosomes, which will allow researchers to reconstruct them by surface modification. These efforts will likely lead to novel disease‐related biomarker discovery, purification tagging, and targeted drug transfer for clinical applications in the future.

## Graphical abstract

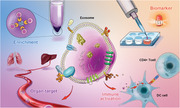

## 1 INTRODUCTION

Lipid‐wrapped extracellular vesicles (EVs) were mentioned in the scientific literature as early as the early 1980s.^1^, ^2^ Since those first studies on the reticulocyte maturation process involving the release of small vesicles, much attention has been focused not only on the process of EV release but also on their sources of release. EV morphology, content, and potential applications of EVs have also been put under the spotlight of investigation. In recent years, exosomes have been identified to be one type of EV, which range from 30 to 100 nm and are secreted by a variety of cells into the surrounding extracellular space or blood. Under the electron microscope, it is apparent that exosomes have a disc‐shaped 3D structure, rather than a spherical structure one might imagine. Exosomes can be found in various body fluids, for example, blood, urine, ascites, and saliva,^3^, ^4^, ^5^, ^6^, ^7^ or in conditioned culture medium of in vitro of cultured cell lines.^5^, ^8^ They contain nucleic acids, lipids, and proteins, all of which may serve as important exosome‐specific biomarkers or specific glycan‐related disease biomarkers.^9^, ^10^ Because of favorable in vivo kinetics of exosomes, high delivery capacity, innate targeting properties, and low immunogenic potentiality, harvesting, and ex vivo manipulation of exosomes shows great promise as a drug delivery system.^11^ Thus, much current research in biology is devoted to study these “sugar‐coated bullets” of tumor: tumor‐derived exosome; that is, completely deciphering the complex structure and function of exosomes to use them in innovative ways to solve difficult biological problems.

## 2 GLYCOSYLATION AND ITS IMPORTANCE IN NORMAL CELLULAR FUNCTION AND PATHOLOGY

**FIGURE 1:**
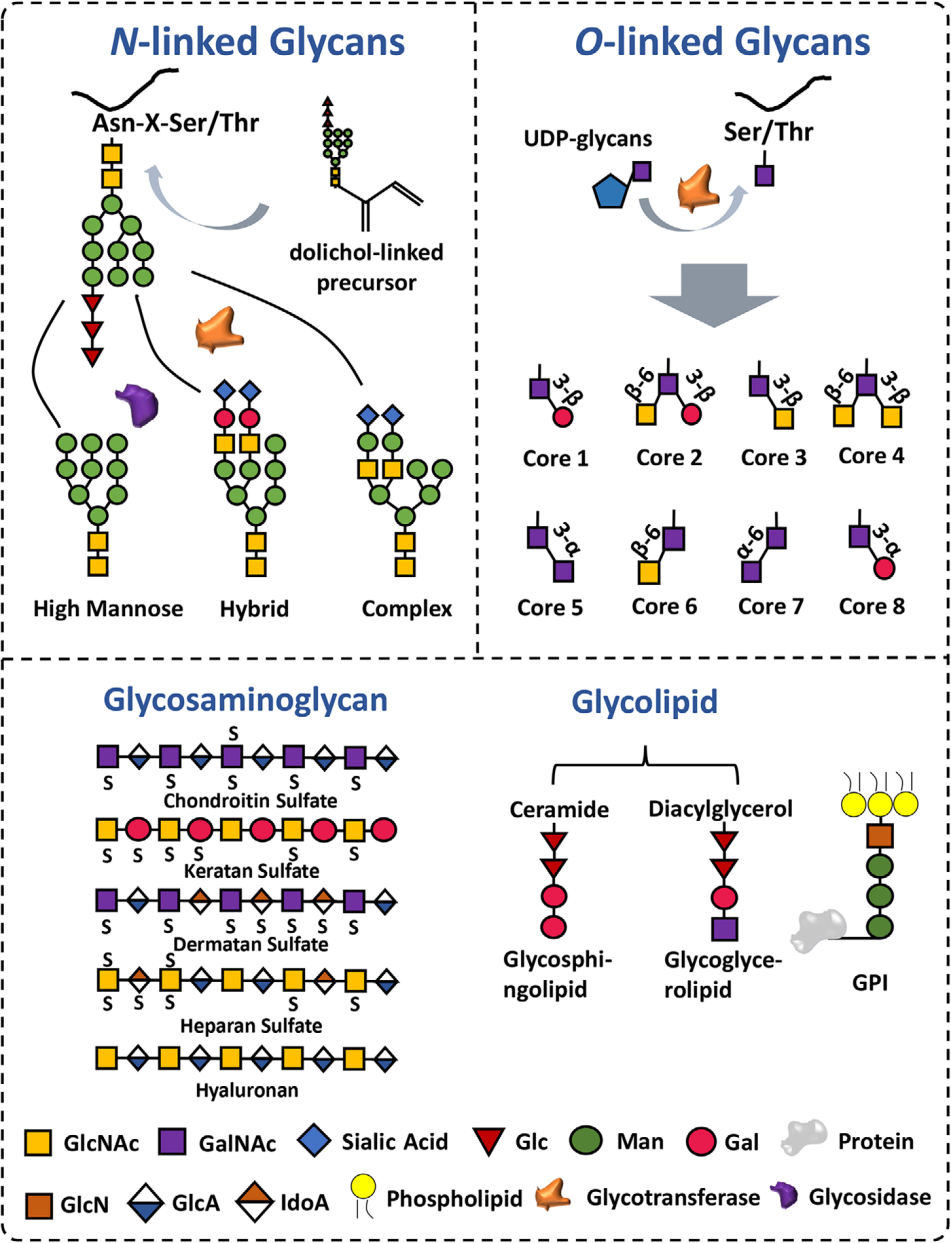
Graphical representation of major glycosylations. Glycans bind to Asn‐X‐Ser/Thr sequon generates three main subtypes of *N*‐glycans: high mannose, complex, and hybrid‐type. Glycans bind to Ser/Thr generates eight *O*‐glycans cores. Glycans bind to ceramides and diacylglycerol generates glycosphingolipids and glycoglycerolipids, respectively. GPI consists of a phosphoethanolamine moiety and a glycan core phospholipid tail, the former connects proteins while the later anchors to the membrane. Proteoglycans are synthesized by a core protein and covalently bound glycosaminoglycan, and glycosaminoglycanis composed of duplicated disaccharide units

Research on EVs reveals an important role of glycans in EV function, and it is clear now that a full understanding of exosome biology and potential applications requires deep and broad knowledge of exosome glycosylation. Glycosylation is one of the most important post‐translational modifications (PTMs), being ubiquitous in all forms of life (Figure 1). Simply put, glycosylation is a process that links carbohydrates to substrates, for example, proteins, lipids, etc., and often glycosylation modulates critical protein functions. In general, the primary protein glycosylation patterns can be divided to two groups: *N*‐linked glycosylation and *O*‐linked glycosylation. Categorization into the two groups is based on the different glycan motifs, structures, and binding site of monosaccharides involved. *N*‐glycans are covalently connected to proteins at asparagine sites. While proteins are constructed in the endoplasmic reticulum (ER), *N*‐linked glycosylation starts by transfer of a dolichol‐linked oligosaccharide precursor to an Asn‐X‐Ser/Thr sequon. The oligosaccharide chain gets further processing in the ER and Golgi apparatus through a process involving glycosyltransferase and glycosidase. This step generates three main subtypes of *N*‐linked glycosylation: high mannose‐type, complex‐type, and hybrid‐type. Complex‐ and hybrid‐type structures may possess several “branches,” known as “antennary.”^12^

In contrast to the *N*‐linked glycosylation process, in *O*‐linked glycosylation, the binding site of a monosaccharide is at the serine and threonine residues of a protein; this is in the glycan core. Unlike with *N*‐glycans, the *O‐*linked glycosylation process that is triggered by glycosyltransferases directly links a monosaccharide to protein, without the requirement of a lipid‐linked precursor formation. Eight different types of glycan core structures are produced after several monosaccharides link to protein, and these structures are further modified by the addition of more sugars to construct an integrated glycoprotein. The most prominent of the *O*‐linked glycosylated glycoproteins is mucin, which is secreted mostly by epithelial cells.^12^ Mucinis, a key *O*‐linked glycosylated glycoprotein needed to lubricate and protect epithelial cells. Mucin production increases during infection, which forms a critical mucus layer, most prominently in the respiratory and digestive tract. Mucin is involved in immune responses and pathogen clearance in the respiratory and digestive systems.^13^ While this mucin production is normal, sometimes the production of mucin is associated with tumor progression, promoting the proliferation, metastasis, adhesion, and drug resistance of many kinds of malignancies through regulating some key signaling pathways.^12^, ^14^, ^15^

Besides the main glycosylation forms mentioned above, other types of glycosylation are also found in mammalian cells that consist of glycolipid, glycosylphosphatidylinositol (GPI), and proteoglycans. Glycosphingolipids and glycoglycerolipids are two types of glycolipid, with a monosaccharide typically binding to ceramide and diacylglycerol, respectively. Glycolipids are situated in membranes, primarily being involved in cell‐cell, cell‐pathogen recognition, and balancing membrane protein physiology. GPI is one specific glycosylation structure that anchors proteins to the membrane. The structure of GPI is characterized by a phosphor ethanolamine moiety, this connects proteins, and a glycan core phospholipid tail which anchors it to the membrane. Disruptions in GPI‐anchored proteins (GPI‐Aps) are reported to be involved in diverse diseases, with mutation of GPI‐anchor genes mainly causing neurological diseases.^16^ The loss of GPI‐Aps are the cause of paroxysmal nocturnal hemoglobinuria.^17^

GPI‐anchored proteins are also in a key position to influence signal transduction in malignancies. For example, Zhang et al. confirmed that GPI‐anchored CD109 downregulates TGF‐β1 signaling and enhances EGF signaling in glioblastoma cells.^18^ This finding is important, because EGF signaling amplification is detected in approximately 40% of primary glioblastomas, suggesting that GPI‐Aps may be a prime target for treating glioblastomas.^18^

Proteoglycans are heavily glycosylated and are synthesized by a core protein and at least one covalently joined glycosaminoglycan chain. Glycosaminoglycan is product of repeating‐connect disaccharide units, for example, hyaluronan, dermatan sulfate, keratan sulfate, chondroitin sulfate, and heparan sulfate.^12^ Proteoglycans are a major component of the extracellular matrix, where they are involved in building up fibrous matrix proteins like collagen.

Although up to this point, we have focused on common forms of glycosylation, some rare types of glycosylation should also be mentioned. Among these are *O*‐Fuc, *O*‐Glc, and *O*‐GlcNAc on epidermal growth factor (EGF)‐like repeats. Thrombospondin type repeats are glycosylated by *O*‐Fuc, and α‐dystroglycan is glycosylated by *O*‐Man. A notable type of glycosylation is *C*‐linked glycosylation, which links sugars to proteins through an uncommon carbon–carbon bond. It is probable that these specific types of glycosylation, albeit not as common, likely have specific influence on certain proteins.^12^

## 3 METHODS TO CHARACTERIZE GLYCOSYLATION

Since the structures of glycans are complicated, profiling and characterizing global glycosylations of a certain target type, for example, exosome, cell, or even tissue, is a huge challenge. There are two main goals with respect to glycan analysis: (1) glycan structure determination, which is included in glycomics, a subset of glycobiology; and (2) precisely locating glycosylation sites. The latter is part of glycoproteomics, a subdivision of proteomics that identifies, catalogs, and characterizes proteins containing carbohydrates resulting from PTMs.^19^ In research on exosomal glycans and glycosylation, researchers have focused more on glycomics.

**FIGURE 2:**
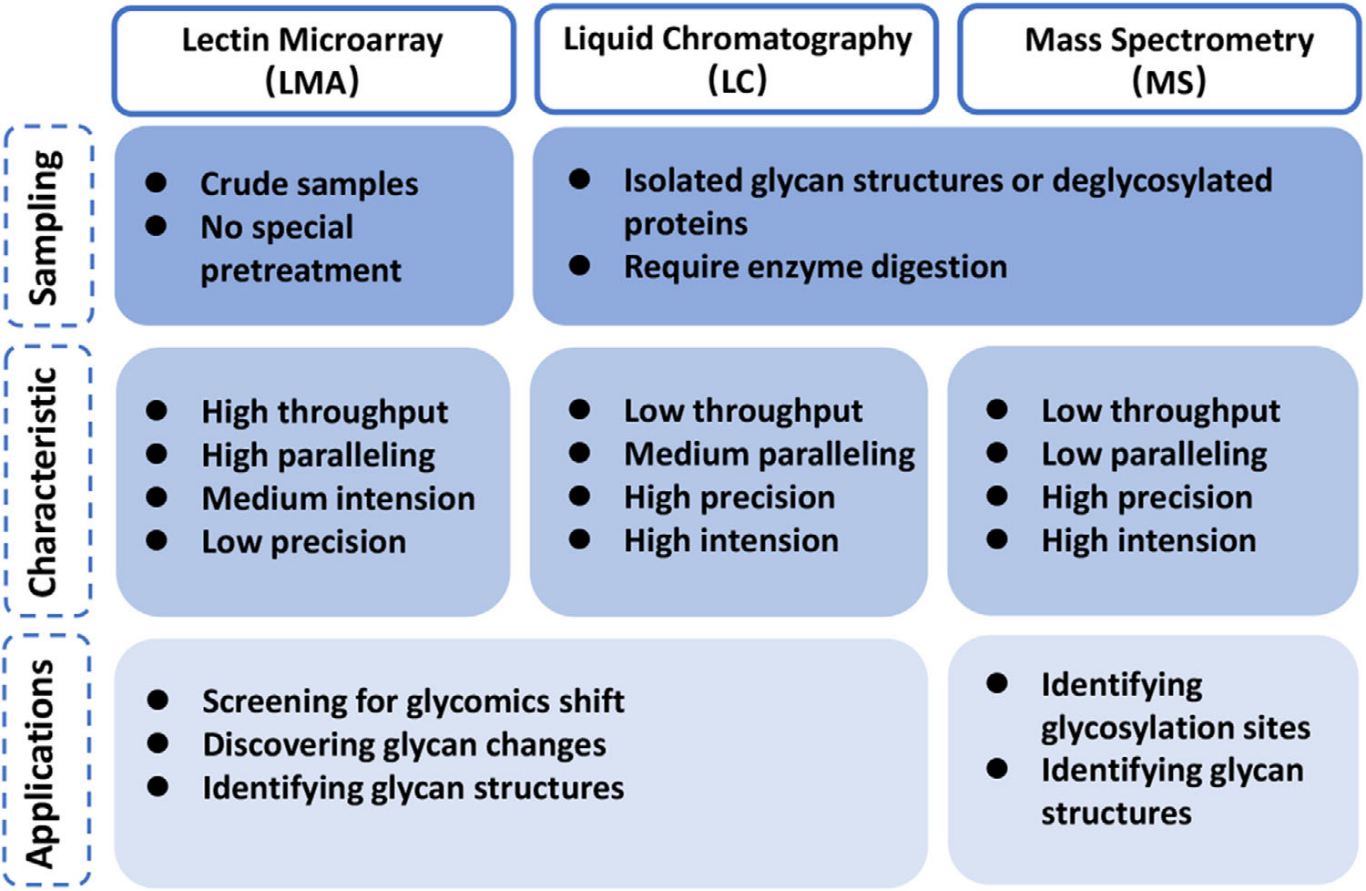
The advantages and characters of the three main methods decoding the structures of glycans

Due to technological advancements in the past decades, many methods and strategies have been developed and refined to decode glycosylation structure. Prominent among these, are advances in mass spectrometry (MS), liquid chromatography (LC), and lectin microarray (LMA) technologies (Figure  2). MS‐based methods include matrix‐assisted laser desorption ionization MS,^20^ electrospray ionization‐mass spectrometry,^21^ and tandem MS^22^ to reveal the structure of a certain glycans. Chromatographic methods, including gas chromatography^23^ and high‐performance liquid chromatography,^24^ are also efficient tools to determine glycan structure. When interference of isomeric structures is a possibility, these methods are more likely to be used in combination with LC‐MS,^19^, ^24^ a technique that combines the physical separation capabilities of LC with the mass analysis capabilities of MS. Detaching a glycan from a biological sample is typically the first step of structural characterization. Glycan separation is done by LC, and derivatization of glycans increases the efficiency of separation by MS.^25^ This is especially the case for methylation, which enhances the ionization of the tested glycan.

Another decoding technique involves lectins, which are a group of proteins primarily obtained from plants, bacteria, fungi, or sometimes animals.^26^ Lectins bind and recognize glycan motifs with high affinity.^26^ Compared to MS and LC, which identify glycan motifs by their molecular weight, lectin recognition identifies certain carbohydrates by their types and structures. In contrast to MS and LC, release of the glycan from the sample is not required when preparing the sample for the lectin‐recognize process. In most cases, lectins serve as the probe in a lectin blot, affinity chromatography, or microarrays, just as with antibodies. It is possible for LMAs to recognize total glycan information of cells or subcellular components in a single assay. Capable of high‐throughput, rapid and parallel detection, LMAs have other significant advantages in screening and comparing even slight shifts in glycosylation structures between samples of various types, for example, cells, proteins, or exosomes.

## 4 GLYCOSYLATION OF EXOSOMES CAN SERVE AS BIOMARKERS OF DISEASE

**FIGURE 3:**
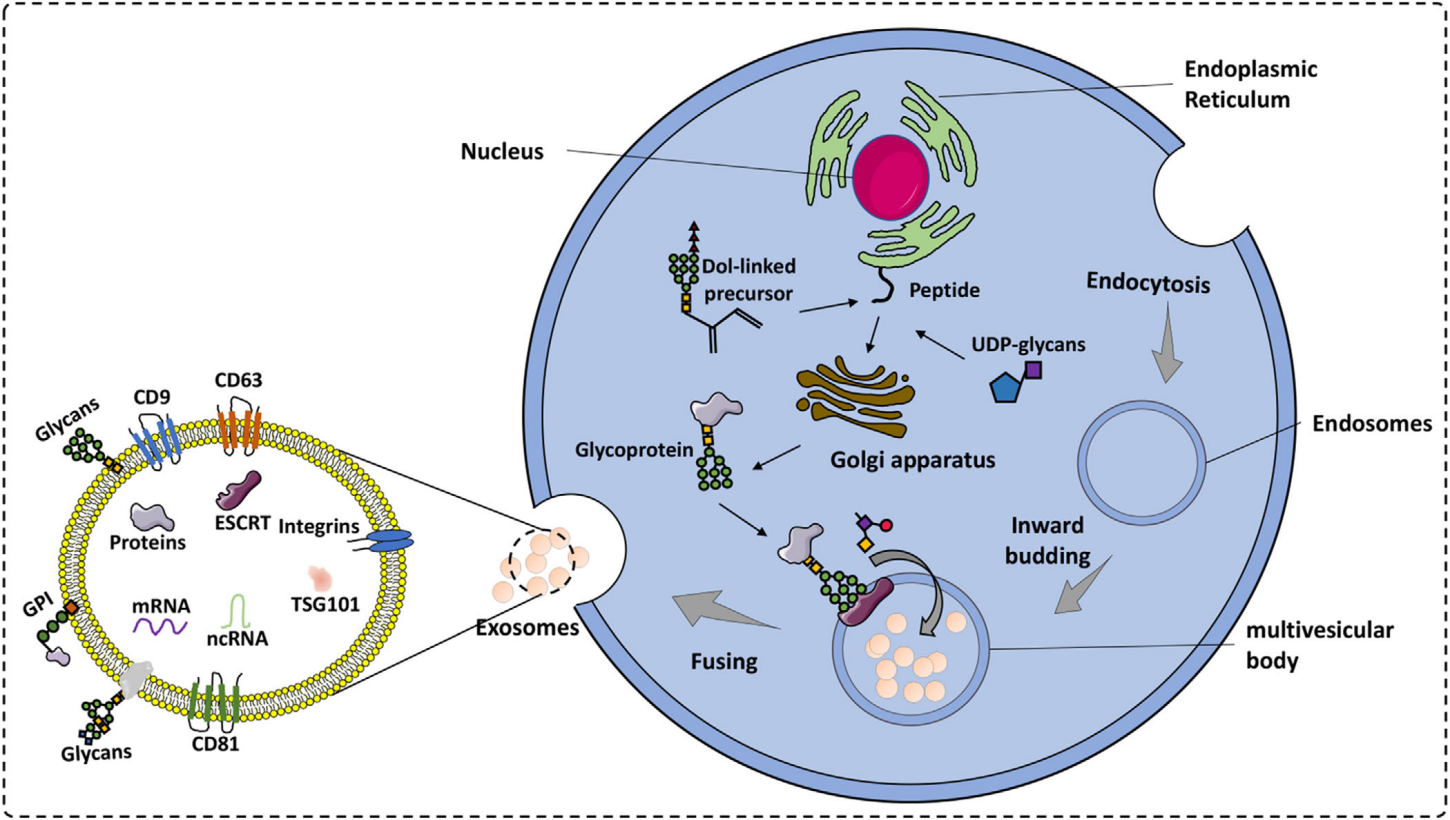
The processes of the biogenesis and glycosylations of exosomes. Exosomes formation starts with endocytosis generating endosomes. Then, inward budding of endosomes membrane formation of ILVs in MVBs, and finally, MVBs fuse with cell membrane and release exosomes.Glycosylated components are recognized by the endosomal‐sorting complex required for transport (ESCRT) and selected into exosomes in the process of ILVs formation

Understanding this application requires one first to understand the basic biology of exosome formation and secretion (Figure 3). To release exosomes, several cellular steps need to be completed. Exosome biogenesis starts with endocytosis. This involves inward budding, or invagination, of the cell membrane, resulting in endosomes. Formation of ILVs in multivesicular bodies (MVBs) occurs next by inward budding of endosomes membrane, and finally, MVBs are transported to the cytoplasma where they fuse and release exosomes into surrounding body fluids.^27^, ^28^

Many components of exosomes are heavily glycosylated.^8^, ^29^ Glycosylated components are recognized by the endosomal‐sorting complex required for transport (ESCRT), which functions in separating out selected cargos into exosomes during ILV formation. Liang *et al* demonstrated in Sk‐Mel‐5 cells that manipulation of glycosylation levels of proteins through genetic engineering can affect their recruitment into exosomes.^30^ Roucourt et al showed also that syndecan, a kind of proteoglycan, combines with syntenin‐1 and ALIX to form a complex involved in the biogenesis and cargo loading of exosomes.^31^

**FIGURE 4:**
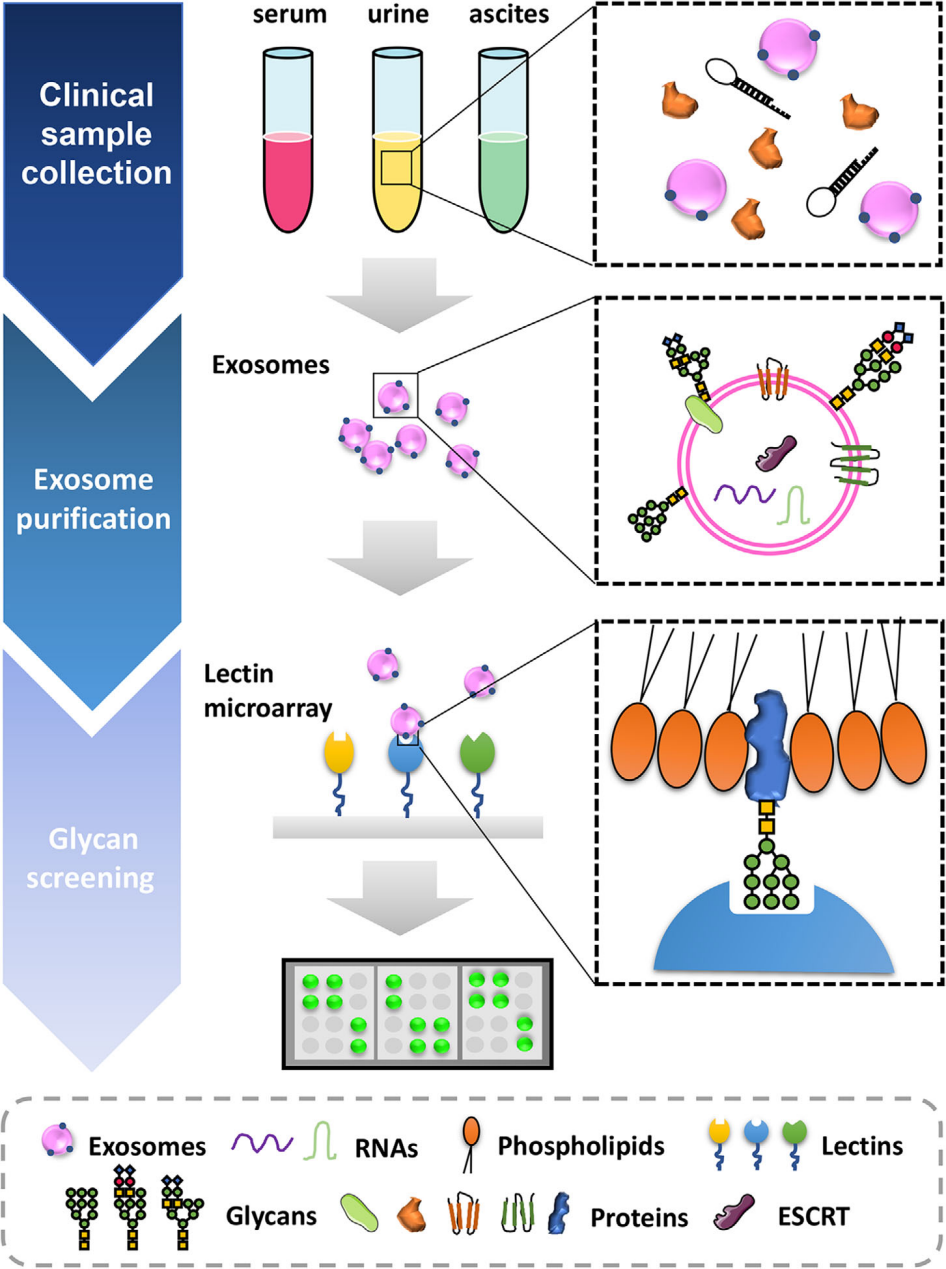
Glycosylation profile of exosomes can serve as biomarkers of disease states. Generally, clinical samples are collected from body fluids such as serum, urine, or ascites. Then exosomes in the samples are purified and detected using a lectin microarray designed specifically to recognize glycosylation in exosomes. The lectin microarray reveals disease‐related shifts in the glycosylation of structures in exosomes, allowing identification of particular disease states in patients donating the samples

As glycosylation patterns of exosomes reflect the patterns of their cells of origin, it is not surprising then that glycosylation patterns of exosomes from tumor cells and nontumor cells differ markedly.^32^ This difference can be exploited for use as the basis of a disease biomarker. Table 1 lists some biomarkers that are targets of exosome glycosylation. Although the surface glycosylation profiles of exosomes are conserved to some extent (ie, rich in sialic acid, mannose, complex *N*‐linked glycans),^8^, ^9^ their patterns also overlap well with those of their host cells.^33^ In essence, therefore, exosomes can be thought of as “representatives” of their parent cells by virtue of their surface glycosylation profile.^33^ Hence, the glycosylation pattern of exosomes can serve as an exquisitely specific and detectable biomarker to help identify patients with certain diseases like cancer (Figure 4).

**TABLE 1:** Selected biomarker targets of exosome glycosylation

Source	Molecule/target	Disease	References
Urine	Urinary vesicle‐associated PSA extraction ratio	PCa	[^38^]
Urine	Tetra‐antennary glycans structure	PCa	[^39^]
Urine	Binding profile of lectins	ADPKD	[^34^]
Urine	Biantennary structure	Galactosemia	[^40^]
Urine	Leucine‐rich α‐2‐glycoprotein	NSCLC	[^41^]
Blood	*O*‐GlcN acylation level of protein	CRC	[^43^]
Blood	α‐2‐HS‐glycoprotein	NSCLC	[^45^]
Blood	MUC1	NSCLC	[^46^]
Ascites	CD24	Ovarian Carcinoma	[^48^]
Ascites	LGALS3BP	Ovarian Carcinoma	[^24^,^50^]
Ascites	CD133 (prominin‐1) and glycosylation level	Pancreatic Cancer	[^6^]

ADPKD, autosomal dominant polycystic kidney disease; CD24, cluster of differentiation 24; CRC, colorectal cancer; LGALS3BP, protein coding for Galectin‐3‐binding protein, MUC1: Mucin 1 cell surface associated; NSCLC, non‐small cell lung cancer; PSA, prostate‐specific antigen; PCa, prostate cancer.

Exosomes are found in various body fluids. Of these, urine is the most accessible, since it can be obtained in a relatively noninvasive way. Gerlach et al demonstrated that exosomes from urine of healthy individuals possess similar glycan composition, implying that exosomal glycan compositions that differ from this normal healthy signature are indicative of the presence of pathology.^34^ Since most urinary exosomes are secreted by cells that line the urinary tract, it is thought that these exosomes reflect the disease state of the urinary system, providing proxy information about disease states of the kidney, prostate, and bladder.^4^, ^34^, ^35^

One well‐known exosomal biomarker is prostate‐specific antigen (PSA), which is a heavily glycosylated protein. Its levels in urine or blood are used widely as a glycoprotein biomarker for screening prostate cancer (PCa) in clinical settings.^36^ However, use of PSA lacks sensitivity and specificity, leading to many misdiagnoses and unnecessary treatment of patients with false positive diagnoses.^37^ For this reason, Vermassen et al developed a new method for enhancing the sensitivity and specificity of PSA‐based tests. They extracted PSA‐associated vesicles with *n*‐butanol, determining the resulting PSA concentration, and finally calculated a ratio comprising total PSA concentration after vesicle‐associated PSA extraction and total PSA concentration before vesicle‐associated extraction. This “urinary vesicle‐associated PSA extraction ratio” is elevated in patients with PCa and is associated with biantennary core‐fucosylation.^38^ When combined with serum PSA values, this approach is more accurate in diagnosing PCa than PSA levels obtained from a single source.^38^

In other work, Julius et al reported that the glycan profile of urinary exosomes have distinct differences in patients without PCa (non‐PCa), patients with indolent PCa, and patients with aggressive PCa.^39^ For example, the glycans of urinary exosomes in indolent PCa are primarily characterized by a bisecting GlcNAc‐hybrid structure, while those in aggressive PCa are mainly characterized by a tetra‐antennary glycan structure.^39^

Urinary exosomes have potential for use not only in diagnosing urinary system disorders, but also nonurinary system disorders. In their LMA analysis of surface glycosylation of urinary exosomes from patients with autosomal dominant polycystic kidney disease (ADPKD), Gerlach et al identified a significant difference in the lectin‐binding profiles of AIA, PA‐I, NPA, RCA‐I, AAL, and GS‐I‐B4 for exosomes obtained from patients with ADPKD and those obtained from healthy individuals.^34^ The glycoprotein profiles of urinary exosomes in patients with galactosemia^40^ and in patients with non‐small cell lung cancer (NSCLC) also differ compared to those in healthy individuals.^41^ Since most metabolic products eventually are excreted into the urinary system, exosomes in the urine could also indicate pathological changes not only of the urinary system, but also of other organ systems and tissues.

Another area in which using exosomal glycosylation profiles as a basis for detecting cancer is early‐stage colorectal cancer (CRC), which is clinically challenging to identify. Until recently, the occult blood test (OBT) was the gold standard for detecting CRC. However, patients with CRC often miss an optimal therapeutic window, because CRC is not detected due to lack of obvious signs and/or insufficient sensitivity of OBT or other biomarkers like carcinoembryonic antigen.^42^ Chaiyawat et al reported that certain proteins in exosomes from a metastatic CRC cell line had higher levels of *O*‐GlcNAcylation than those from a nonmetastatic cell line.^43^ Those proteins belong to the cadherin superfamily cell adhesion and ATPase superfamily of proteins.^43^ As these kinds of differences in glycosylation are thought to be closely related to tumor metastasis, information on the glycosylation state of exosomal proteins can be used for clinical tumor, node, metastasis staging of CRC in conjunction with CT scans, MRI, and endosonography.^42^

Regarding NSCLC, Niu et al compared exosomes from the sera of 125 patients with NSCLC and 46 healthy donors, and found that exosomes from the patients express significantly more α‐2‐HS‐glycoprotein (AHSG) having a remarkable biantennary structure and presence of extracellular matrix protein 1 (ECM1).^44^, ^45^ These two biomarkers in combination have better diagnostic efficiency than that of AHSG or ECM1 alone.^45^ Along the same lines, Pan et al observed upregulation of mucin 1 (MUC1) in exosomes from patients with NSCLC compared to that in healthy individuals.^46^

Another source of exosomes having diagnostic potential is serous fluid in the peritoneal cavity. Tumor metastasis to the peritoneum, angiogenesis, metabolite, or tumor cells themselves can facilitate ascites.^47^ Thus, ascites that contain exosomes from these sources can possess abundant disease‐relevant information. For example, in ovarian carcinoma patients, Runz et al identified glycoprotein CD24 in EpCAM‐expressing exosomes from ascites.^48^, ^49^ CD24 is a highly *O*‐glycosylated mucin‐like glycoprotein closely associated with aggressive and advanced stage malignancies.^48^, ^49^ Escrevente et al found that exosomes derived from ovarian carcinoma patients also contain a characteristic glycosylated protein, LGALS3BP, whose *N*‐glycans bears complex glycans having di‐, tri‐, and tetra‐antennary structures and high mannose type glycans. These glycans are also highly related to poor prognosis, metastasis, and drug resistance.^24^, ^50^

Pancreatic cancer‐related exosomes also are found in ascites. Sakaue et al discovered that exosomes derived from pancreatic patients express a glycoprotein (CD133) biomarker for pancreatic cancer.^6^ Importantly, they demonstrated that the degree CD133 glycosylation is associated with patient prognosis, such that higher degrees of glycosylation (eg, sialic acids, GlcNAc, mannose, etc.) translate to better prognosis.^6^

As mentioned above, urine is undoubtedly an ideal source of exosomes for assessing exosomal glycosylation, as urine is easily accessible. Such analysis can be used for detecting various kinds of dysfunction in diverse organs and for diagnoses. Another source of exosomes is blood. As blood circulates throughout the body, compared to exosomes in urine, exosomes in blood should carry more information useful for diagnosing more disorders. Yet another source of exosomes is ascites. Ascite‐related exosomes show greater potential for use in diagnosing diseases of the peritoneum than do exosomes derived from urine or blood.

## 5 POSSIBILITY OF USING EXOSOME GLYCOSYLATION FOR EXOSOME PURIFICATION

As mentioned, exosome glycosylation has diagnostic potential as a biomarker for many different kinds of diseases. Despite their importance, however, a major challenge is isolating high‐quality exosomes in sufficiently large quantities from various kinds of clinical samples. Overcoming this challenge require purification of exosomes form biological samples. Presently, differential ultracentrifugation (UC) is still the most commonly used method for purification.

Differential UC involves a series of repeated steps that separate suspended particles on the basis of sedimentation rate. Different amounts of centrifugal force are applied to a liquid sample to progressively eliminate cell debris, proteins, and other undesired contaminants, ultimately yielding a final pellet of concentrated exosomes.^35^, ^51^ However, exosomes obtained by differential UC are insufficient in purity, and this method requires excessive time and expensive specialized equipment. Because of these obstacles, researchers have used other methods to obtain high‐quality exosomes, like density‐gradient UC, which produces higher purity and quantity of exosomes. Ultrafiltration is another method, which uses nanomembranes to concentrate exosomes. The method is time saving and uses a simple protocol. Size exclusion chromatography leverages the known variances in size of exosomes and contaminants. It effectively removes any contaminants that could interfere with separation of various exosomes, while reducing the separation time to less than 10 min.^52^, ^53^, ^54^ As noted by Freitas et al these different isolation approaches may result in various subgroups of exosomes, having diverse diameters, concentrations, protein profiles, and glycosylation profiles.^55^

The importance of lectin‐glycan‐binding exosome purification was highlighted by Echevarria et al, who demonstrated that the unique affinity and specific lectin binding to exosomes, not to contaminants, can be the basis for effectively removing impurities, leaving concentrated exosomes.^26^ Kosanovic et al showed this by using Concanavalin A lectin to immobilize exosomes on plates for a binding assay.^51^ They found dose‐dependent lectin‐glycan binding that can be blocked with mannose. Evidence from transmission electron microscopy showed that this reversible binding takes place in the suspension phase.

**FIGURE 5:**
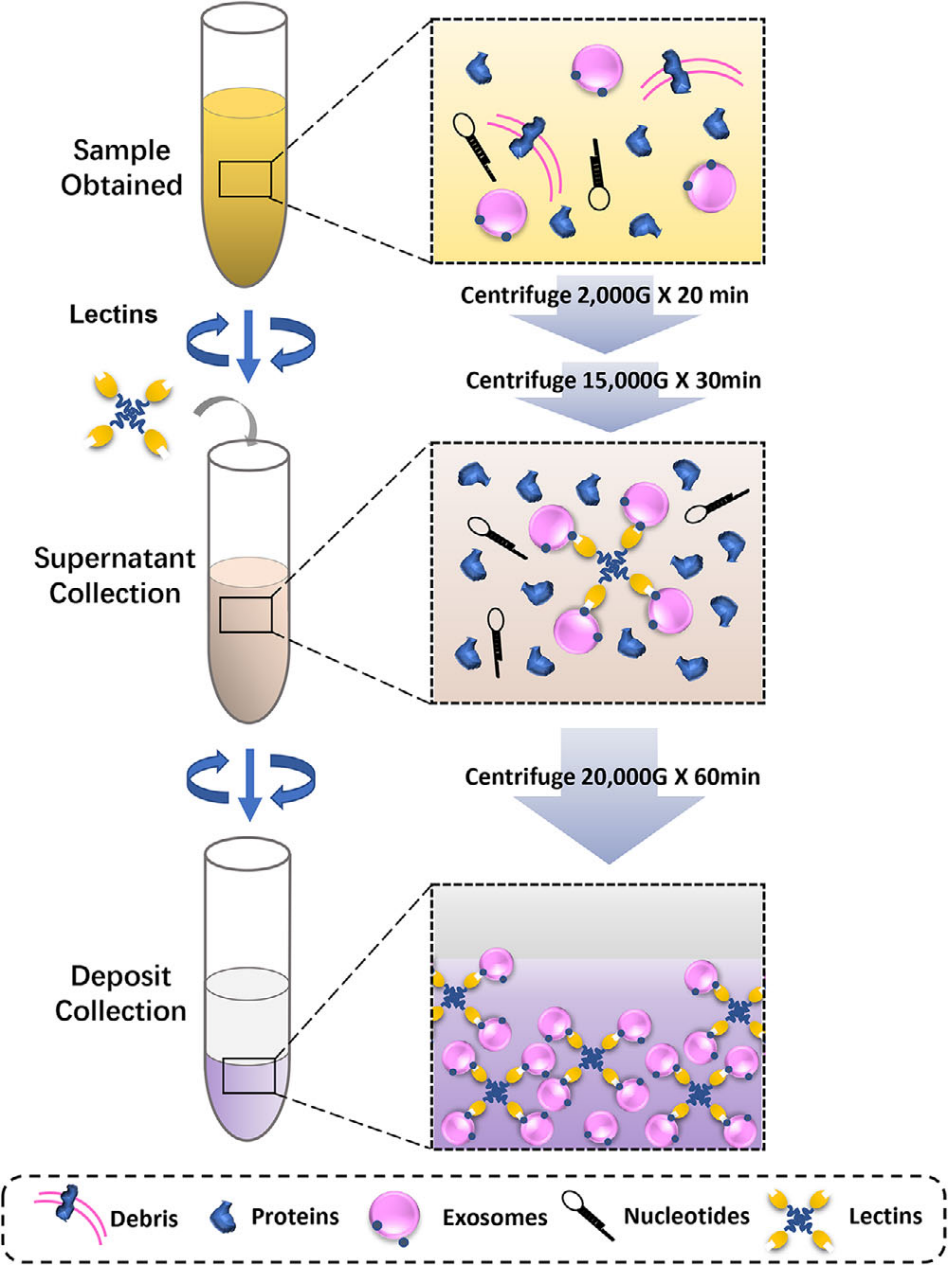
Glycosylation profile of exosomes can be used as a way to purify exosomes from body fluids. First, the clinical sample undergoes two centrifugations to eliminate proteins and cell debris. Second, the resulting supernatant, which contains exosomes and a small amount of contaminants, is collected and mixed with certain lectins that bind specifically to glycosylated compounds located on the exosomes. Finally, the lectin‐labeled exosomes can be separated easily by centrifugation

In most cases, lectin‐glycan‐binding is combined with UC (Figure 5). Constituent proteins or cell debris in clinical samples are eliminated by UC, then selected lectins are suspended in the collected supernatant for binding and assembling glycosylated moieties located in exosomes. After lectin binding, exosomes can be separated easily with another round of UC.^5^ In a comparative study, Gerlach et al captured exosomes by binding with lectins or antibodies.^56^ With each separation technique, they measured average yields of exosomes by total protein content. Average yield with lectins was significantly lower than with antibodies. However, when they compared vesicle counts using nanoparticle tracking analysis, the number of exosomes yielded by four of the six lectins was much higher than by antibodies.^56^

Lectin‐glycan‐binding microarray has emerged as a useful tool for high‐yield, high‐quality exosome separation. It is a simple, rapid, and high‐throughput separation technology that is superior in both time and process to differential UC. Its use is definitely advantageous in biomarker discovery and further analyses of exosome glycosylation modification. This purification protocol solves the problem of low recovery of exosomes needed to proceed reasonably to clinical applications.

## 6 SURFACE GLYCAN MODIFICATION FOR DIRECTING EXOSOMES TO TARGET CELLS

**FIGURE 6:**
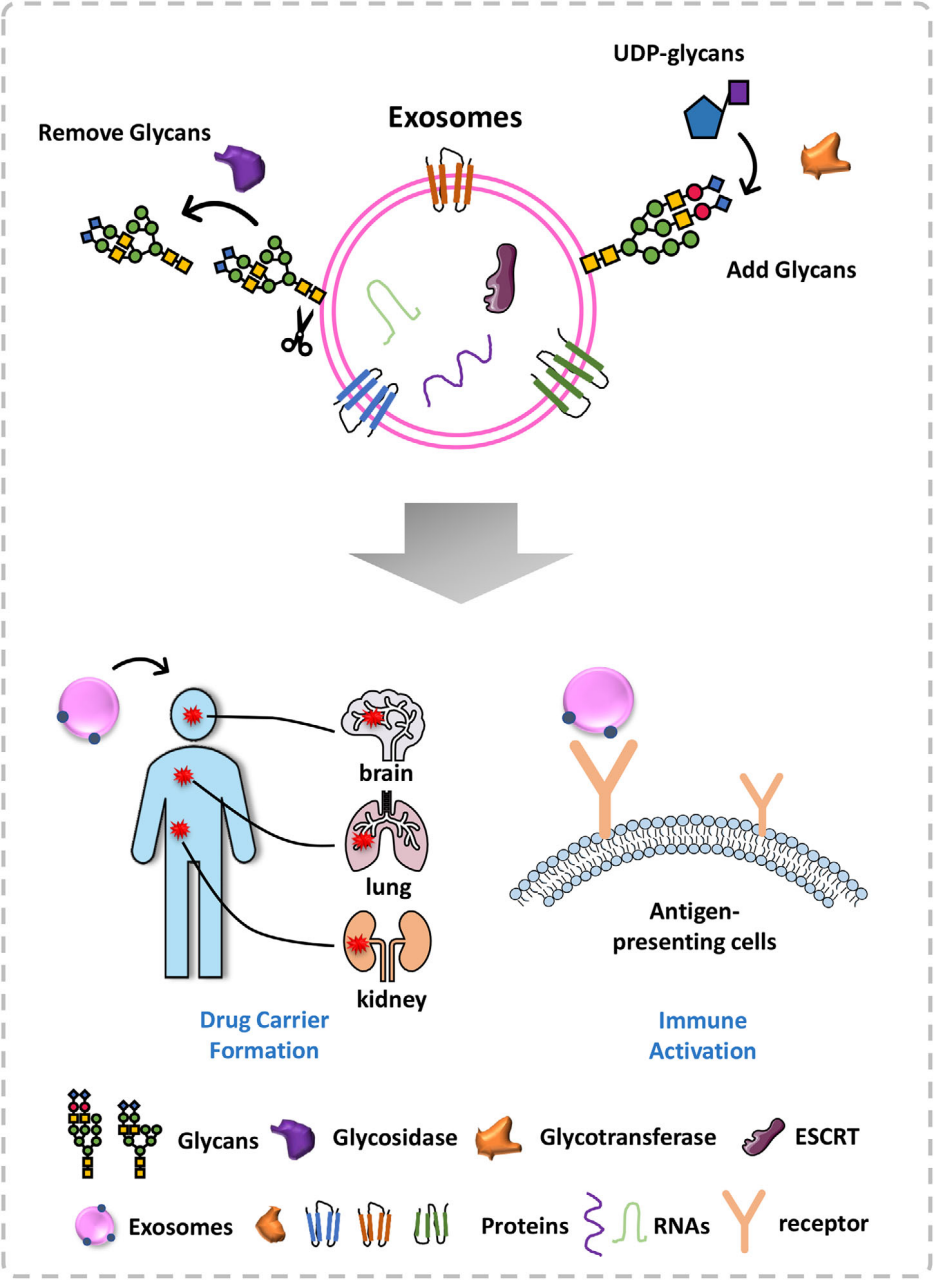
Glycosylation profile of exosomes can be used as a tool to selectively target therapeutics to specific tissues. Through ex vivo manipulation of exosome glycosylation (remove or add glycans), manipulated exosomes can be used to activate antitumor immunity or to build a drug carrier that can then be reintroduced to the body to selectively target a tissue with the exosome‐carrying drug; UDP‐glycan, uridine 5′‐diphosphate glycan

Since surface glycosylation plays an important role in mediating exosome targeting and absorption, researchers have attempted to manipulate the glycosylation profiles of exosomes with the goal of artificially directing exosome traffic.^29^, ^57^ Surface glycan modification refers to the removal, addition, or change of glycan motifs on the surface of exosomes (Figure 6). Many glycosylation‐related enzymes have been employed as tools to accomplish modifications. For example, *N*‐glycosidase F (commonly known as PNGase F) is typically used to completely remove *N*‐linked glycan structures. PNGase F acts on the covalent bond between asparagine and GlcNAc. Endo H and neuraminidases are also used frequently. These cleave high mannose and sialic acid structures, respectively.^8^ However, because no specific enzyme is available to remove *O*‐linked glycans, these are usually removed chemically, for example, with β‐elimination or hydrazinolysis.^19^ It is a prevailing viewpoint that exosomes play a central role in tumor invasion.^58^ Whether altering the glycosylation pattern of exosomes would improve the tumor status of cancer patients remains to be demonstrated. Nonetheless, manipulating the glycosylation characteristics of tumor‐derived exosomes is currently a hot topic in cancer research.

Since exosomes exhibit similar tumor‐specific glycan markers as their host cells of origin,^33^ this feature has been exploited in attempts to specifically activate the immune system using tumor cell‐derived exosomes. This inherent targeting characteristic and tropism feature could be exploited to selectively activate the immune system against specific kinds of tumors through cell‐derived exosomes, while avoiding off‐target effects. This characteristic could also be leveraged to incorporate drugs in exosomes, and then selectively deliver the drugs to their intended targets. This can be more complicated than appreciated at first glance. For example, Dusoswa et al found that the glycan motifs on glioblastoma‐derived exosomes were unsuitable tumor antigens that could not activate the immune system properly in vivo.^59^ To overcome this limitation, they removed the immunosuppressive glycans and inserted a high‐affinity glycoprotein ligand for antigen‐presenting cells (APCs). Modified exosomes showed promise as an effective endogenous cell‐free antigen source for APCs in the context of antitumor therapeutics. These modified exosomes significantly relieved immunosuppression and successfully activated antitumor immunity.^59^

On the other hand, exosomes are ideal vehicles for drug delivery in vivo because of their small volume and low immunogenicity.^60^, ^61^, ^62^ These two advantages make exosomes attractive for drug delivery, cargo loading, and remote transfer to target tissue. Glycosylation might play important roles in the latter. Nishida et al reported glycosylation has a suppression function in exosome uptake.^63^ Removal of surface glycans of exosomes derived from human breast cancer cells enhanced the uptake of these exosomes in vivo by human umbilical vein endothelial cells (HUVECs).^63^ Similarly, Royo et al removed sialic acid from mouse liver‐derived exosomes, which led to an obvious increase in number of exosomes, their reach, and assembly in both lungs.^64^ They confirmed that glycosylation could mediate transfer of exosomes in vivo.^64^ In addition, Choi et al reported that they could modify the surface of bovine serum‐derived exosomes with mannose together with polyethylene glycol, successfully increasing the number of exosomes delivered to dendritic cells and lymph nodes in vivo.^64^, ^65^

Hence, the modification of proper glycan motifs on the surface of exosomes serves as a supplement for canonical tumor therapy, whereas the addition of certain glycans could activate antitumor immunity and govern exosome transport (Figure 6). Undoubtedly, the modification of exosome surface glycans will be a promising strategy for exosome targeting and transfer.

## 7 DISCUSSION

Although exosomes were discovered decades ago, scientific interest in them has accelerated only in the last decade or so. They are now a prime research focus in basic biology, clinical diagnostics, and “smart” drug delivery. Their various and diverse features make them attractive to biologists and clinicians for many reasons. For example, exosomes have the ability to penetrate physiological barriers like the blood–brain barrier, and the nature of their lipid membrane structure prevents their contents from being diluted and digested in physiological fluids. These, among other reasons, make them scientifically useful not only for improving our understanding of cell‐cell communication, but also as potential delivery vehicles for pharmaceuticals and as accessible markers of disease states. As mentioned, emerging pieces of evidence also suggest that exosome glycosylation has the brilliant prospect of being useful for novel therapeutic applications. Hence, given that much is yet to be learned about exosomes, it can easily be concluded that the glycosylation of exosomes should gain only more research attention in the future. However, there are still caveats to be aware of that may dampen the frenzy of enthusiasm about exosome applications.

First, although the analysis of glycosylation has resulted in delightful progress in screening exosomes related to disease biomarkers, the majority of these studies have been done with cell or animal models rather than with clinical samples from patients. This limitation may make current results less meaningful for clinical applications. Hence, it is necessary that more research be done using clinical samples, and extensive clinical trials are required in future studies. Second, as secreted exosomes derive from multiple organs and tissues, the quantity and quality of exosomes in clinical samples vary widely. For example, lung cancer‐related exosomes can be detected in three body fluids: pleural effusion, serum, and urine. Therefore, the optimal source of exosomes related to different diseases needs to be addressed before this approach to body‐fluid diagnostics can be translated into useful clinical applications. More tissue comparative studies on the efficacy of using exosomes as biomarkers from diverse sources are also necessary. Third, when considering the glycosylation of exosomes, researchers have paid more attention to glycoproteins, with related technologies being developed and optimized primarily for them.^22^, ^66^ However, glycoproteins are not the only component that is glycosylated in exosomes. It must be pointed out that glycolipids also play an important role in the biological functions of exosomes. For instance, glycosphingolipids, one type of glycolipids in exosomes, are reported to bind amyloid‐β (Aβ) peptide in the brain.^67^ This finding has contributed to a meaningful and improved understanding of Aβ levels and possible ways to treat the symptoms of Alzheimer disease.^67^ Thus, in the future more research attention should be paid to elucidating the role glycolipids, proteoglycans, and even monosaccharides play in exosome structure and function.

In summary, much progress has been made in understanding the glycosylations of tumor‐derived exosomes: the “sugar‐coated bullets of tumors.” Surface glycosylation in exosome‐related study is a novel, hot research area, as multiple research groups have published their work on the analysis and modification of the surface glycosylation profiles of exosomes in disease‐related biomarker discovery, purification tagging, and drug targeting and transfer to affected tissues. These fundamental discoveries, and the diversity of applications borne out of them, indicate great potential for utilizing aspects of exosome glycosylation in future clinical applications. Hence, we firmly believe that a deep understanding of the fundamentals of surface glycosylation of exosomes will further broaden potential clinical applications for exosomes and open new windows for using exosomes in innovative ways to solve difficult biological problems.

## AUTHORS' CONTRIBUTIONS

Shanyi Lin wrote the article. Shumin Zhou and Ting Yuan reviewed and edited the manuscript. All authors approved the final version of the manuscript.

## CONFLICT OF INTEREST

The authors declare that they have no conflict of interest.
